# Factors associated with under-five mortality in Rwanda: An analysis of the Rwanda Demographic and Health Survey 2020

**DOI:** 10.1371/journal.pgph.0003358

**Published:** 2024-06-18

**Authors:** Mgole Eliud Mkama, Mark Momoh Koroma, Xiaofen Cheng, Jindan Zhang, Duo Chen, Lushi Kong, Bei Li

**Affiliations:** 1 School of Health Management, Southern Medical University, Guangzhou, China; 2 Department of Epidemiology, School of Public Health, Southern Medical University (Guangdong Provincial Key Laboratory of Tropical Disease Research), Guangzhou, China; JIPMER PSM: Jawaharlal Institute of Post Graduate Medical Education and Research Department of Preventive and Social Medicine, INDIA

## Abstract

Despite global and Rwandan progress in reducing under-five mortality, the risk of children dying before their fifth birthday persists, necessitating intensified research on determinants. Thus, this study analyzed the birth history data to shed light on the underlying causes of under-five mortality in Rwanda. The study is a secondary analysis of data sourced from the 2020 Rwanda Demographic and Health Survey (RDHS) cross-sectional design. Using SPSS, the data was cleaned, recoded, and weighted, with descriptive and inferential statistics applied. The dependent variable was the child’s living status, while independent variables included socio-demographic, media exposure status of mothers, child, and environmental factors. A total of 10267 under-five children of all interviewed mothers were included in the final analysis, of which 12.3% (1260) died. Maternal age (25–34 years: AOR = 1.514, 95% CI = 1.130–2.029, p = 0.005; 45+: AOR = 13.226, 95% CI = 9.253–18.905, p<0.001), occupational status (agricultural workers and other services), and three or more births within five years (AOR = 1.895, 95% CI = 1.433–2.508, p<0.001) significantly increase the risk of under-five mortality. Conversely, maternal education (primary: AOR = 0.821, p = 0.023; secondary: AOR = 0.533, p<0.001; higher: AOR = 0.365, p = 0.010) and higher wealth indexes (middle: AOR = 0.743, p = 0.001; rich: AOR = 0.612, p<0.001), as well as current breastfeeding (AOR = 0.524, 95% CI = 0.455–0.603, p-value <0.001), are associated with lower under-five mortality. Child sex significantly impacts under-five mortality (AOR = 0.873, 95% CI = 0.770–0.991, p = 0.035), favoring females over males. Conversely, multiple birth type children face higher under-five mortality (AOR = 3.541, 95% CI = 2.727–4.599, p<0.001) compared to singletons. Children in the northern (AOR = 1.478, 95% CI = 1.086–2.011, p = 0.013) and eastern (AOR = 1.470, 95% CI = 1.097–1.971, p = 0.010) regions are more susceptible to mortality compared to those in the central (Kigali) region. Additionally, under-five mortality is higher when using water from tanks and other sources (AOR = 2.240, 95% CI = 1.471–3.411, p<0.001) than piped water. This study identifies crucial factors linked to under-five mortality, underscoring the importance of prioritizing them in interventions to enhance Rwandan under-five survival rates.

## Introduction

Under-five mortality remains a critical lens through which the health landscape of a nation and its population is evaluated [1]. The World Health Organization (WHO) defines under-five mortality as the overall number of child deaths occurring within the initial five years of life for every 1000 live births [2]. While there has been significant global progress, the mortality rate has decreased by almost 60% over the past 30 years [3]. It remains a major burden in sub-Saharan Africa and South Asia. In 2020, over 80% of the 5 million under-five deaths occurred in these regions, with sub-Saharan Africa contributing the highest number (2.8 million) [3, 4]. This concerning concentration of under-five mortality is further highlighted by the fact that the global rate stagnated at 5 million deaths in 2021, with a significant proportion attributed to preventable or treatable conditions, malnutrition, and various socioeconomic challenges [5, 6]. Moreover, many researches have provided insights into the mother, child, and socio-demographic factors contributing to under-five mortality in sub-Saharan African countries (SSA) [7, 8]. Most of these factors were linked to poverty and need to be investigated with a customized approach to eliminate each country’s causes.

Rwanda, an SSA country striving to achieve middle-income status by 2035, has shown significant progress in addressing under-five mortality. Data compiled by the Inter-agency Group for Child Mortality Estimation (UNICEF, the World Health Organization, United Nations, and World Bank) reveals a remarkable decline in the Rwanda under-five mortality rate, dropping from approximately 341 in 1994 to about 40 children in 2021 [9]. This translates to a 70% decline between 2000 and 2011, positioning Rwanda among the leading low-income countries in SSA on track to achieve MDG4 by 2015 [10, 11]. This positive trend can be attributed to various health initiatives that improved geographic access by increasing the number of primary healthcare facilities, reducing financial barriers, and implementing community-based health insurance [12, 13]. Noteworthy collaborations, such as the Rwanda government’s partnership with health organizations to establish the White Ribbon Alliance (WRA), have been instrumental. The WRA is dedicated to reducing maternal and newborn mortality rates, with a significant focus on ensuring the well-being of children up to their fifth birthday [14]. However, to achieve the 2030 Sustainable Development Goals (SDGs), Rwanda must intensify efforts to enact efficacious strategies addressing inequities and decreasing death rates among children under five [15–18]. This could involve comprehensive research on the various determinants of under-five mortality, utilizing demographic and health surveys. The DHS data is a valuable resource for understanding various health indicators, as it is nationally representative and appropriate for determining factors associated with under-five mortality in countries where it is conducted.

The Demographic Health Survey utilizes the birth recode file to capture data on every child born to interviewed women, providing comprehensive estimates of under-five mortality for all children born to eligible women. This study employs the 2020 Rwanda Demographic Health Survey (RDHS.) Birth Recode dataset to identify factors associated with under-five mortality in Rwanda. Through thoroughly examiningthis dataset, we aim to unravel the complex web of socioeconomic, maternal, child-related, and environmental determinants influencing under-five mortality. The findings will inform recommendations to policymakers, non-governmental organizations (NGOs), and other stakeholders, facilitating the implementation of effective interventions to reduce nationwide under-five mortality rates.

## Methods

### Study setting

The study was conducted in Rwanda, a nation classified as a low-middle-income country with an estimated population of around 12 million individuals [19]. Geographically, Rwanda is divided into five provinces: Central, East, West, North, and South, with each province further comprising districts. This decentralized administrative framework significantly contributes to Rwanda’s achievements in enhancing maternal and child health (MCH) metrics. The Rwandan health system is organized into a tiered network of facilities, providing accessible care at various levels:

### Health posts

These are located in villages and staffed by Community Health Workers (CHWs), offering essential maternal and child health (MCH) services such as antenatal care, postnatal care, childhood vaccinations, and family planning. A total network of 406 health posts plays a vital role in ensuring access to basic healthcare in rural areas [20].

### Health centers

These are located in the in districts and provide a broader range of Maternal and Child Health (MCH) services. They are staffed by nurses, midwives, or clinical officers capable of managing complex deliveries and childhood illnesses and offering additional services. There are 495 health centers, forming the backbone of the health infrastructure, which heavily relies on a tiered network of primary healthcare facilities dedicated to MCH service delivery.

### District hospitals

As the name suggests, they are located in the districts and serve as referral points for complicated cases requiring specialized care within the Rwandan health system. This structured approach ensures that Maternal and Child Health (MCH) services are geographically accessible to most Rwandans, particularly those in rural settings [21]. However, despite its effectiveness, the system faces several challenges that can impede service delivery and contribute to child mortality. In total, the Rwandan health system comprises 8 national referral hospitals, 4 provincial-level hospitals, and 35 district-level hospitals.

### Study design

This study conducts a secondary analysis of data from the 2020 Rwanda Demographic and Health Survey, a cross-sectional survey implemented every 5 years as part of the global Demographic and Health Surveys project to monitor and evaluate population, health, and nutrition programs”.

The sample design was based on the 2012 Rwanda Population and Housing Census (RPHC) conducted by the National Institute of Statistics of Rwanda (NISR) [19]. It is a cross-sectional design that involves two-stage approaches to facilitate the estimation of key indicators at various levels, including national, urban, rural, provincial, and district. The first stage involved selecting 500 clusters from the 2012 RPHC enumeration areas (EAs), which comprised 112 urban and 388 rural areas. The second stage included the systematic selection of households from lists compiled during a household listing operation carried out in these chosen EAs between June and August 2019. A total of 13,000 households were included, with 26 households sampled from each cluster [19]. Weighting factors were applied to the data to ensure representative results at the national level.

### Source of data

Data was acquired by developing a concept note, enabling access to RDHS data via the Measure DHS program’s website (www.measuredhs.com), which collects data in more than 90 countries, primarily in low- and middle-income nations. This collaborative initiative involved the National Institute of Statistics of Rwanda (NISR) and the Ministry of Health (MOH) to improve the health outcomes of Rwanda’s population. The extensive survey collected demographic and health data from a nationally representative sample of women aged 15–49 [19]. Data collection spanned from November 9, 2019, to July 20, 2020, with a temporary halt of over two months due to a nationwide lockdown in response to the COVID-19 pandemic. The BR file containing data about under-five children was used and is accessible at https://dhsprogram.com/data/availabledatasets.cfm.

### Sample size

The data included 10267 children within the age of five years ever born to an interviewed mother. Our analysis specifically included all births within the five years prior to the survey. We included all living children within five years of age and all instances of under-five mortality, excluding any deaths of children older than five years.

### Dependent variable

**Fig 1:**
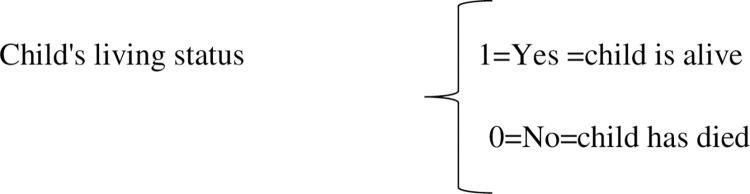
Final recoded values for child living status.

The dependent variable in this study, originally coded as "Child’s alive," was recoded to represent the child’s living status for children between 0–59 months old. This variable was obtained based on the binary response of interviewed mothers about the survival status of their children under five. The binary response to the question (child’s living status) was "No," which was coded as "0," and "Yes" coded as "1". Since the outcome of interest was death/mortality, the variables were transformed and recoded inversely. Thus, the final recoded values for child living status are as shown in Fig 1.

### Independent variables

The independent variables were selected based on prior research that has shown an association between these factors and under-five mortality [22–26]. They were categorized into socio-demographic characteristics, environmental, media exposure status of mothers, and child factors. Socio-demographic factors constitute marital status, breastfeeding, mothers’ age, pregnancy status, number of childbirths in the past five years preceding the survey, the total number of children born, and educational level. Environmental factors were the residence region and the delivery place in a private or public facility. In contrast, the media exposure status of mothers includes the frequency of watching television, listening to the radio, reading newspapers/magazines, owning mobile phones, and using the internet. Finally, the child factors entail sex, birth size, and birth type. This categorization was based on the conceptual framework developed by Mosley and Chen for child survival in developing countries [27].

### Data processing and statistical analysis

Data analysis was conducted using SPSS version 26 statistical software. The data was first cleaned recoded, and the weight (WGT) variable was generated (v005/1,000,000). The weight variable was applied throughout the analysis to consider the possibility of uneven sampling and guarantee that all strata were adequately represented. Moreover, multiple filters were applied to ensure that only death within five years was included in the final analysis since the birth recode file was used. Descriptive statistics were then employed, and frequency tables were generated to describe the proportions of all explanatory variables. Subsequently, inferential statistics was employed, and a Chi-square test was conducted to determine the association between the independent and the dependent variables. The binary logistic model included all variables that exhibited significant association at p-value <0.05. The univariate P-values, 95% confidence interval (CI), and crude odds ratio (COR) were displayed. The final logistic model included all independent variables associated with under-five mortality at P ≤ 0.25. The results were displayed as adjusted odds ratios (AOR), confidence intervals (CI), and P-values. Independent variables that exhibited significance at p-value<0.05 of the final multivariable regression were used to explain the causes of under-five mortality.

### Ethics statements and consent to participate

The data collection in the Rwanda Demographic and Health Survey (RDHS) was granted by the Institutional Review Board of the Rwandan Ministry of Health. Each participant voluntarily consented to the primary data collection of the RDHS. Our study obtained written permission to access the dat

aset from MEASURE DHS, the organization tasked with overseeing demographic health surveys globally, and from the ICF Institutional Review Board.

## Results

### Distribution characteristics and the factor associated with under-five mortality

A total of 10267 children within the age of five years whose mothers were interviewed were included in the final analysis. Of these, 12.3% (1260) of children were reported to have died below the age of five. The distribution characteristics are described as follows:

A significant portion of mothers (47.2%) fall within the 25–34 age group and have primary education (65.8%). Most mothers (78.4%) lack bank accounts, while a substantial number work in agriculture (41.6%) or unskilled labor (24.1%). Media exposure is limited, with low rates of newspaper reading (18.3%), radio listening (76.0%), TV viewing (39.3%), and internet usage (9.1%). Children present a nearly even gender distribution (50.8% male, 49.2% female) with high health insurance coverage (79.6%), and more than half of them were being breastfed (62.4%). Geographically, the study population is predominantly rural (83.3%), with the highest density in the East region (27.1%) as exhibited in Table 1.

**Table 1:** Distribution characteristics of participants.

CHARACTERISTICS	FREQUENCY 10267	PERCENTAGE 100%
**SOCIO-DEMOGRAPHIC FACTORS**		
**Maternal Age**		
15–24	1388	13.5
25–34	4842	47.2
35–44	3678	35.8
45+	358	3.5
**Education Attainment**		
No Education	1319	12.8
Primary	6757	65.8
Secondary	1796	17.5
Higher	395	3.8
**Occupational Status/Types**		
Not Working & Household domestic	2161	21.1
Agricultural workers or Employers	4270	41.6
Services	1162	11.3
Skilled workers	203	2.0
Unskilled workers	2470	24.1
**Bank or Financial account**		
No	8049	78.4
Yes	2218	21.6
**Wealth Index**		
Poor	4553	44.3
Middle	1978	19.3
Rich	3735	36.4
**Marital Status**		
Never in union	784	7.6
Currently in union/living with a man	8619	83.9
Formerly in a union/living with a man	864	8.4
**Current Breastfeeding**		
No	3861	37.6
Yes	6405	62.4
**Birth in last 5 years**		
1	5858	57.1
2	3864	37.6
3+	544	5.3
**Health Insurance Type**		
No	2091	20.4
Yes	8176	79.6
**MEDIA EXPOSURE STATUS OF MOTHERS**		
**Reading Newspaper**		
Not at all	8387	81.7
Less than a week	1327	12.9
At least once per week	552	5.4
**Frequency of Listening Radio**		
Not at all	2461	24.0
Less than once a week	2045	19.9
At least once a week	5761	56.1
**Frequency of Watching TV**		
Not at all	6230	60.7
Less than once a week	2453	23.9
At least once a week	1583	15.4
**Use of Internet**		
Never	9331	90.9
Yes, the last 12 months	771	7.5
Yes, before the last 12 months	165	1.6
**Owns a Mobile Telephone**		
No	5792	56.4
Yes	4474	43.6
**CHILD FACTORS**		
**Sex of Child**		
Male	5211	50.8
Female	5055	49.2
**Type of Birth**		
single birth	9923	96.7
multiple births	343	3.3
**ENVIRONMENTAL FACTORS**		
**Region**		
Central (Kigali)	1346	13.1
South	2065	20.1
West	2496	24.3
North	1581	15.4
East	2778	27.1
**Residence**		
Urban	1710	16.7
Rural	8557	83.3
**Source of Drinking Water**		
Piped water	4378	42.6
Well Water	568	5.5
Spring Water	4046	39.4
River, Lake, and Pond water	875	8.5
Rain Water	51	0.5
Tank and other sources of Water	182	1.8
Bottled Water	166	1.6

### Factors associated with under-five mortality

Generally, all the distributive characteristics were significantly associated with under-five mortality at a significance of 0.05 (Table 2)

**Table 2:** Factors associated with under-five mortality.

CHARACTERISTICS	Alive N = 9007 %		Mortality N = 1260 %		P VALUE
	Number	100%	Number	100%	
**SOCIO-DEMOGRAPHIC FACTORS**					
**Maternal Age**					**<0.001**
15–24	1328	14.7	60	4.8	
25–34	4494	49.9	349	27.7	
35–44	2990	33.2	688	54.6	
45+	195	2.2	163	12.9	
**Education Attainment**					**<0.001**
No Education	1050	11.7	269	21.3	
Primary	5864	65.1	893	70.9	
Secondary	1709	19.0	87	6.9	
Higher	384	4.3	11	0.9	
**Occupational Status/Types**					**<0.001**
Not Working & Household domestic	1987	22.1	175	13.9	
Agricultural workers or Employers	3640	40.4	630	50.0	
Services	1043	11.6	119	9.4	
Skilled workers	187	2.1	16	1.3	
Unskilled workers	2149	23.9	321	25.5	
**Bank or Financial account**					**0.001**
No	7015	77.9	1034	82.1	
Yes	1992	22.1	226	17.9	
**Wealth Index**					**<0.001**
Poor	3851	42.8	702	55.7	
Middle	1750	19.4	229	18.2	
Rich	3405	37.8	330	26.2	
**Marital Status**					**<0.001**
Never in union	731	8.1	53	4.2	
Currently in union/living with a man	7566	84.0	1052	83.5	
Formerly in a union/living with a man	710	7.9	155	12.3	
**Current Breastfeeding**					**<0.001**
No	3207	35.6	654	51.9	
Yes	5800	64.4	605	48.1	
**Birth in last 5 years**					
1	5116	56.8	742	56.8	
2	3437	38.2	427	33.9	
3+	453	5.0	91	7.2	
**Health Insurance Type**					**<0.001**
No	1770	19.7	321	25.5	
Yes	7237	80.3	939	74.5	
**MEDIA EXPOSURE STATUS OF MOTHERS**					
**Reading Newspaper**					**<0.001**
Not at all	7290	80.9	1097	87.1	
Less than a week	1208	13.4	119	9.5	
At least once per week	509	5.7	43	3.4	
**Frequency of Listening Radio**					
Not at all	2094	23.2	367	29.1	**<0.001**
Less than once a week	1790	19.9	255	20.2	
At least once a week	5123	56.9	638	50.6	
**Frequency of Watching TV**					
Not at all	5392	59.9	838	66.5	
Less than once a week	2154	23.9	299	23.7	
At least once a week	1460	16.2	123	9.8	
**Use of Internet**					**<0.001**
Never	8108	90.0	1223	97.1	
Yes, the last 12 months	740	8.2	32	2.5	
Yes, before the last 12 months	159	1.8	5	0.4	
**Owns a Mobile Telephone**					**<0.001**
No	4974	55.2	818	64.9	
Yes	4033	44.8	442	35.1	
**CHILD FACTORS**					
**Sex of Child**					**0.021**
Male	4534	50.3	678	53.8	
Female	4473	49.7	582	46.2	
**Type of Birth**					**<0.001**
single birth	8782	97.5	1142	90.6	
multiple births	225	2.5	118	9.4	
**ENVIRONMENTAL FACTORS**					
**Region**					**<0.001**
Central (Kigali)	1252	13.9	94	7.5	
South	1805	20.0	260	20.6	
West	2215	24.6	281	22.3	
North	1360	15.1	221	17.5	
East	2374	26.4	404	32.1	
**Residence**					**<0.001**
Urban	1570	17.4	139	11.0	
Rural	7437	82.6	1121	89.0	
**Source of Drinking Water**					**<0.001**
Piped water	3929	43.6	449	35.6	
Well Water	481	5.3	88	7.0	
Spring Water	3514	39.0	532	42.2	
River, Lake, and Pond water	726	8.1	149	11.8	
Rain Water	44	0.5	7	0.6	
Tank and other sources of Water	148	1.6	34	2.7	
Bottled Water	165	1.8	1	0.1	

### Determinants of under-five mortality in Rwanda

The correlates of under-five child mortality in Rwanda based on crude (unadjusted) and adjusted logistic regressions are presented in Table 3. Generally, the univariate logistic model exhibited significance for all the variables associated with under-five mortality at the Chi-square analysis. However, the final multivariable logistic model revealed that maternal age, educational attainment, occupational status, wealth index, current breastfeeding, number of births within five years, sex of the child, the type of birth, region, and source of the drinking water were significant determinants of under-five mortality of all births history of interviewed mothers and were presented below.

**Table 3:** Determinants of under-five mortality in Rwanda based on the DHS 2019–20 birth recode data.

CHARACTERISTICS	COR	95% CI		P-VALUE	AOR	95% CI		P-VALUE
**SOCIO-DEMOGRAPHIC FACTORS**								
**Maternal Age**								
15–24	1				1			
25–34	1.706	1.290	2.258	**<0.001**	1.514	1.130	2.029	**0.005**
35–44	5.058	3.858	6.631	**<0.001**	4.328	3.231	5.797	**<0.001**
45+	18.441	13.240	25.686	**<0.001**	13.226	9.253	18.905	**<0.001**
**Education Attainment**								
No Education	1				1			
Primary	0.595	0.511	0.692	**<0.001**	0.821	0.693	0.973	**0.023**
Secondary	0.200	0.155	0.257	**<0.001**	0.533	0.393	0.723	**<0.001**
Higher	0.113	0.061	0.208	**<0.001**	0.365	0.169	0.789	**0.010**
**Occupational Status/Types**								
Not Working & Household domestic	1				1			
Agricultural workers or Employers	1.968	1.649	2.347	**<0.001**	1.293	1.059	1.578	**0.012**
Services	1.292	1.011	1.651	**0.040**	1.408	1.077	1.842	**0.012**
Skilled workers	0.975	0.572	1.660	0.924	1.211	0.688	2.133	0.508
Unskilled workers	1.699	1.399	2.063	**<0.001**	0.980	0.785	1.224	0.861
**Bank or Financial account**								
No	1				1			
Yes	0.768	0.660	0.895	**0.001**	0.946	0.794	1.127	0.534
**Wealth Index**								
Poor	1				1			
Middle	0.717	0.611	0.841	**<0.001**	0.743	0.619	0.891	**0.001**
Rich	0.531	0.462	0.610	**<0.001**	0.612	0.498	0.754	**<0.001**
**Marital Status**								
Never in union	1				1			
Currently in union/living with a man	1.916	1.439	2.550	**<0.001**	0.992	0.724	1.357	0.958
Formerly in a union/living with a man	2.998	2.159	4.165	**<0.001**	1.264	0.883	1.809	0.201
**Current Breastfeeding**								
No	1				1			
Yes	0.512	0.454	0.576	**<0.001**	0.524	0.455	0.603	**<0.001**
**Birth in last 5 years**								
1	1				1			
2	0.856	0.755	0.972	0.016	1.099	0.945	1.277	0.221
3+	1.386	1.092	1.758	0.007	1.895	1.433	2.508	**<0.001**
**Health Insurance Type**								
No	1				1			
Yes	0.716	0.624	0.821	**<0.001**	0.901	0.772	1.052	0.187
**MEDIA EXPOSURE STATUS OF MOTHERS**								
**Reading Newspaper**								
Not at all	1				1			
Less than a week	0.656	0.538	0.801	**<0.001**	0.934	0.748	1.168	0.550
At least once per week	0.564	0.411	0.775	**<0.001**	0.932	0.648	1.340	0.703
**Frequency of Listening Radio**								
Not at all	1				1			
Less than once a week	0.814	0.686	0.967	0.019	0.885	0.731	1.071	0.208
At least once a week	0.711	0.619	0.816	**0.000**	1.042	0.878	1.236	0.641
**Frequency of Watching TV**								
Not at all	1				1			
Less than once a week	0.894	0.776	1.029	0.118	1.096	0.930	1.293	0.274
At least once a week	0.541	0.444	0.660	**<0.001**	1.002	0.778	1.291	0.987
**Use of Internet**								
Never	1				1			
Yes, the last 12 months	0.283	0.197	0.406	**<0.001**	1.019	0.620	1.674	0.941
Yes, before the last 12 months	0.222	0.093	0.526	**<0.001**	0.590	0.239	1.459	0.254
**Owns a Mobile Telephone**								
No	1				1			
Yes	0.665	0.588	0.753	**<0.001**	0.906	0.771	1.064	0.228
**CHILD FACTORS**								
**Sex of Child**								
Male	1				1			
Female	0.871	0.774	0.981	**0.022**	0.873	0.770	0.991	**0.035**
**Type of Birth**								
single birth	1				1			
multiple births	4.043	3.209	5.094	**<0.001**	3.541	2.727	4.599	**<0.001**
**ENVIRONMENTAL FACTORS**								
**Region**								
Central (Kigali)	1				1			
South	1.917	1.498	2.453	**<0.001**	1.213	0.896	1.642	0.212
West	1.690	1.324	2.156	**<0.001**	1.108	0.830	1.480	0.487
North	2.163	1.679	2.787	**<0.001**	1.478	1.086	2.011	**0.013**
East	2.269	1.794	2.869	**<0.001**	1.470	1.097	1.971	**0.010**
**Residence**								
Urban	1				1			
Rural	1.697	1.411	2.040	**<0.001**	0.925	0.725	1.181	0.534
**Source of Drinking Water**								
Piped water	1				1			
Well Water	1.595	1.245	2.044	**<0.001**	1.088	0.824	1.437	0.551
Spring Water	1.324	1.158	1.513	**<0.001**	1.011	0.863	1.184	0.889
River, Lake, and Pond water	1.791	1.464	2.191	**<0.001**	1.083	0.859	1.367	0.500
Rain Water	1.433	0.647	3.174	0.376	1.248	0.536	2.905	0.608
Tank and other sources of Water	2.042	1.392	2.995	**<0.001**	2.240	1.471	3.411	**<0.001**
Bottled Water	0.069	0.012	0.388	**0.002**	0.215	0.037	1.259	0.088

### Maternal socio-demographic determinants

Most maternal socio-demographic factors were significant determinants of under-five mortality in Rwanda among all live births. Generally, maternal age, occupational status/type, and the number of births in the past five years by a mother were more likely to influence an increase in under-five mortality. Educational attainment, wealth index, and current breastfeeding of a child predicted a lower likelihood of a child dying below the age of five.

Maternal age was a significant determinant of childhood mortality in Rwanda. Specifically, children born to mothers with higher age groups had a significantly higher chance of dying below the age of five compared to children born to younger mothers. In particular, children born to mothers aged 25–34 (AOR = 1.514, 95% CI = 1.130–2.029, P-Value = 0.005), 35–44 (AOR = 4.328, 95% CI = 3.231–5.797, P-value <0.001) and 45+ (AOR = 13.226, 95%CI = 9.253–18.905, p-value <0.001) were respectively 1.5, 4.3 and 13.2 times more likely to die below the of five compared to those born to mothers aged 15–24 years old. Similarly, the occupational status or the occupation type of the mother and the number of births given by the mother in the past five years significantly increased the likelihood of under-five mortality in Rwanda. In particular, children who were born to mothers who were agricultural workers (AOR = 1.293, 95% CI = 1.059–1.578, p-value = 0.012) and employees of other services (AOR = 1.408, 95% CI = 1.077–1.842, p-value = 0.012) were more likely to die before their fifth birthday compared to those born to mothers that are not working or only does household domestic chores. Additionally, children whose mothers had three births and above (AOR = 1.895, 95% CI = 1.433–2.508, p-value <0.001) within the last five years preceding the survey were 1.4 times more likely to die before their fifth birthday.

On the other hand, higher educational attainment of the mothers was associated with lesser under-five mortality. Specifically, children whose mothers attained primary school (AOR = 0.821, 95% CI = 0.693–0.973, p-value = 0.023), secondary (AOR = 0.533, 95% CI = 0.393–0.723, p-value <0.001) and higher (AOR = 0.365, 95% CI = 0.169–0.789, p-value = 0.010) were less likely to die below the age of five compared those children whose mothers did not go to school. Moreover, children whose mothers fall in the middle index (AOR = 0.743, 95% CI = 0.619–0.891, p-value = 0.001) and the rich (AOR = 0.612, 95% CI = 0.498–0.754 p-value<0.001) were less likely to die at under-five compared to those born to poor mother. Similarly, the breastfeeding status of a child was a significant influencer of childhood mortality. Generally, children who were currently breastfeeding (AOR = 0.524, 95% CI = 0.455–0.603, p-value <0.001) were 0.5 times less likely to die at under-five compared to those children who were not breastfeeding.

### Media exposure status of mothers

Generally, all the media exposure statuses of mothers as a factor were significantly associated with under-five mortality in the univariate model but not in the multivariable model.

### Child factor

Child factors (sex and birth type) exhibited a significant influence on under-five mortality at both univariate and multivariable levels. In terms of sex, females (AOR = 0.873, 95% CI = 0.770–0.991, p-vaule = 0.035) were 0. 9 times less likely to die below the age of five years compared to their male counterparts. On the other hand, multiple birth type children (AOR = 3.541, 95% CI = 2.727–4.599, p-values <0.001) were significantly 3.5 times more likely to die below their fifth birthday compared to single birth children.

### Environmental factors

Environmental factors exhibited a significant influence on childhood mortality at the univariate level. However, only the region and the type of water source exhibited significance at the multivariable level. Under-five children living in the north (AOR = 1.478, 95% CI = 1.086–2.011, p-value = 0.013) and east (AOR = 1.470, 95% CI = 1.097–1.971, p-value = 0.010) were approximately 1.5 times more likely to die below the age of five compared to children in the central (Kigali).

Similarly, under-five mortality was 2.4 times more likely to occur with drinking water from tanks and other sources (AOR = 2.240, 95% CI = 1.471–3.411, p-value <0.001) than drinking piped water. However, drinking water from rivers, lakes, and ponds exhibited marginal significance.

## Discussion

### Determinants of under-five mortality in Rwanda on the RDHS 2020 births recode data

The study investigates the determinants of under-five child mortality ever born to all interviewed mothers in Rwanda, thus shedding light on significant maternal socio-demographic factors (maternal age, educational attainment, maternal occupation, wealth index, breastfeeding status, number of births in five years), child factors (child’s sex and type of birth), and geographical or environmental factors (region, and drinking water source). The media exposure status of mothers as a factor did not exhibit any significant influence on under-five mortality in Rwanda. These findings provide crucial insights into the multifaceted nature of all under-five mortality recorded by the 2020 RDHS birth history.

### Maternal socio-demographic determinants

#### Maternal age

Maternal age emerged as a significant determinant of under-five mortality in Rwanda. Children born to older mothers face a higher risk of mortality, with a remarkable gradient in risk as maternal age increases. In particular, children born to mothers aged 35–44 and 45+ are 4.33 and 13.23 times substantially more likely to die before the age of five compared to those born to younger mothers, which is similar to findings in Ghana and Kenya [28, 29]. This outcome also aligns with studies from Sierra Leone and aggregated data from recent Demographic and Health Surveys in 30 Sub-Saharan African countries (2010–2019), underscoring the effect of maternal age on child survival [26, 30]. However, our data suggests a stronger association between maternal age and under-five mortality compared to Ghana, with AOR = 11.4 (age 35–49) [28, 29]. There could be different reasons for this finding, which may include the increased risk of pregnancy complications such as gestational diabetes, preeclampsia, and placental problems associated with advanced maternal age that can affect the health of both the mother and the child, potentially leading to adverse outcomes [31]. It is important to note that age has been implicated in under-five mortality differently. For instance, Koroma et al. exhibited that younger mothers aged 15–19 (teen mothers) in Sierra Leone were more likely to lose their children below the age of five compared to old-age mothers. However, our study did not categorize teen mothers separately.

Moreover, the different ages in different countries have different influences based on cultural practices. Hence, age is a crucial determinant of under-five mortality. Implementing targeted health policies and awareness campaigns for the different maternal ages is vital for enhancing maternal and child health and improving under-five survivability.

#### Occupational status

Occupational status or the type of occupation undertaken by the mother also plays a significant role in under-five mortality. Children born to mothers engaged in agricultural work or employed in other services are respectively 1.30 and 1.41 times at an increased risk of under-five mortality, similar to an earlier study in Nigeria [32]. However, the association between occupational status, such as agricultural work, and under-five mortality is less pronounced (OR = 1.30) in our study compared to Nigeria, where it is stronger (OR = 2.68) [32]. The possible reason for the increased risk of under-five mortality among this occupational status group could be explained in terms of the possible insufficient focus on child caregiving by agricultural women and other service providers (sales and manual labor). For example, a study in Nigeria documented that these occupational categories exhibit the lowest rates of childhood vaccination coverage [33], which could make children vulnerable to preventable child diseases. Occupational status and child mortality have yielded mixed outcomes in earlier studies; Hence, occupational status or types should be greatly considered in health planning policies and strategies to target interventions for specific occupational groups to reduce child mortality rates.

#### Number of births in five years

Additionally, the number of births in five years by a mother is associated with an increase in under-five mortality. Children born to mothers with three or more births in this period face an elevated risk of mortality, similar to recent studies conducted in Sierra Leone and Ghana [26, 34, 35]. However, our study exhibited a weaker (AOR = 1.90) association between three births in five years and under-five mortality compared to Sierra Leone (AOR = 8.11) for the same three births in five years [26, 34, 35]. The elevated risk of child mortality with an increase in the number of births could stem from difficulties in providing sufficient care and attention to each child. For example, Khan et al. identify malnutrition, especially in children with low birth size, as a major factor contributing to child mortality [36]. Inadequate care can result in poor or unbalanced nutrition, heightening susceptibility to diseases. Ensuring access to family planning services and maternal education for mothers with higher parity may increase childbirth spacing andchild survival.

#### Maternal education and wealth index

Maternal educational attainment and wealth index are other important determinants of under-five mortality exhibited in our study. Higher maternal education and belonging to the middle and rich wealth index were associated with lower under-five mortality. Children whose mothers have attained primary, secondary, or higher education are, respectively, 0.82, 0.53, and 0.37 times

less likely to die before the age of five compared to children whose mothers did not receive formal education, as supported by recent findings in Sierra Leone, Nigeria, and Tanzania [37, 38]. Children born to mothers with primary educational attainment in our study had a significantly lower risk of under-five mortality (AOR = 0.82) compared to children born to Tanzania mothers with the same educational level attainment (AOR = 0.76) [37, 38]. Mothers with secondary education in Rwanda had a lower risk of under-five mortality for their children (AOR = 0.53) compared to Nigeria (AOR = 0.75). However, Tanzania showed an even greater protective effect (AOR = 0.30) for mothers with the same education level. Similarly, children born to mothers in the middle or rich wealth index categories are less likely to die under the age of five compared to those born to mothers categorized as poor [39]. Our findings suggest that academic attainment and wealth index likely play a crucial role in influencing child health outcomes. Educated mothers may be better equipped to utilize resources, regardless of their income level. They might have increased health awareness, improved healthcare navigation skills, and a stronger voice in decision-making regarding their children’s health. Additionally, wealth can provide access to vital resources such as nutritious food, sanitation facilities, and quality healthcare services, further improving child health prospects. This highlights the potentially synergistic effect of these factors. Mothers with both higher education and greater wealth might be particularly well-positioned to ensure the well-being of their children [37, 38]. This underscores the importance of investing in maternal education to reduce childhood mortality, improve household economic status, and enhance child survival rates.

#### Breastfeeding status

Current breastfeeding status was found to be a significant influencer of under-five mortality. Children who were currently breastfeeding were 0.52 times less likely to die under the age of five compared to those who were not breastfeeding, similar to that exhibited by an early study in Sierra Leone and Nigeria [26, 40]. However, the association of current breastfeeding demonstrated a slightly higher risk of under-five mortality in our study compared to that in Sierra Leone (AOR = 0.20) [37, 38]. There could be several reasons for this finding. Breast milk provides essential nutrients and antibodies that help strengthen a child’s immune system, protecting against infections and diseases [41–43].

Additionally, breastfeeding promotes bonding between the mother and child and the emotional connection, which may positively affect the child’s overall well-being. Access to clean water and proper sanitation, which are common challenges for formula feeding in some regions, might also play a role. It’s a complex interplay of biological, environmental, and socioeconomic factors. This highlights the critical role of breastfeeding in child health and emphasizes the importance of promoting and supporting breastfeeding practices.

#### Media exposure status of mothers

Our study demonstrated that the media exposure status of mothers was significantly associated with under-five mortality in the univariate model. Yet, this association did not reach statistical significance in the multivariable model, contrasting with findings from another study [44]. It suggests that while there may be individual associations between the media exposure status of mothers and under-five mortality when considered in isolation, these factors lose significance when analyzed with other variables. Possible implications could be that the influence of media exposure status of mothers as a factor on under-five mortality might be confounded or influenced by other variables included in the multivariable model. It could also indicate that the relationship is not as direct or substantial when accounting for other relevant factors.

### Influences of child factors on under-five mortality

#### Sex and birth type

The analysis of child factors, specifically sex and birth type, were significant determinants of under-five mortality in Rwanda. These findings are similar to earlier studies conducted in Sierra Leone and Ethiopia using similar DHS [26, 45]. In particular, females exhibited a lower likelihood of under-five mortality compared to males, consistent with studies in Sierra Leone, Ethiopia, and Indonesia [26, 44, 46] and highlighting gender differentials in child health outcomes. The lower risk of female under-five mortality (AOR = 0.87) in our study was almost similar to that in Ethiopia (AOR = 0.84) [26, 45] but was higher than that of Sierra Leone (AOR = 0.68) [26, 45].

This finding could be attributed to complex and multifactorial reasons, such as biological, social, and cultural factors, behavioral differences, and environmental factors, which may vary depending on the specific context and population being studied. For instance, biologically, research suggests that the increased risk of male child mortality may be attributed to their XY chromosomes, which are more susceptible to X-linked recessive disorders than the XX chromosomes in females, resulting in poorer overall health outcomes for males compared to females [47, 48]. Our finding raises questions about potential gender-specific vulnerabilities or protective factors that should be further explored to inform targeted interventions. More importantly, healthcare professionals and policymakers should develop gender-sensitive interventions and public awareness campaigns to address gender-specific vulnerabilities contributing to under-five mortality in Rwanda.

Children born as multiples had a notably higher probability of not surviving past their fifth birthday than those born singletons. This finding is consistent with earlier studies in Sierra Leone and Ethiopia [26, 45, 49]. Our study exhibited a stronger association (AOR = 1.41) between multiple births and under-five mortality compared to Sierra Leone (AOR = 1.41) [26, 45]. However, this association was observed to be weaker in Ethiopia (AOR = 4.755) [26, 45]. The increase in under-five mortality with multiple births could be attributed to factors such as higher rates of premature birth, lower birth weights, increased susceptibility to specific health conditions, and challenges in prenatal development, all of which are more common in multiple pregnancies and can impact the overall health and survival rates of these children [50]. It could also be due to the sharing of limited resources, including food and care provided by the mother, among multiple children. The implications of this finding are crucial for maternal and child health programs, emphasizing the need for specialized care and attention for mothers carrying multiple pregnancies.

### Geographical or environmental factors

#### Region and type of water source

Environmental factors exhibited a significant influence on under-five mortality at the univariate level. However, only the region and the type of water source exhibited significance in the multivariable analysis. Under-five children living in the north and east were more likely to die below the age of five compared to children in the central (Kigali). Similarly, under-five mortality was more likely to occur with drinking water from tanks and other sources compared to drinking piped water [51, 52]. However, drinking water from rivers, lakes, and ponds exhibited marginal significance.

These findings align with other studies in Ethiopia that exhibit regional differences in under-five mortality [45, 53]. The higher under-five child mortality in the north and eastern regions compared to the central area (Kigali) could be attributed to disparities in healthcare access, infrastructure, and education, societal or cultural factors unique leading to differences in healthcare quality, living conditions, and levels of health awareness and practices among communities [54]. Investigating regional under-five child mortality disparities will be necessary to understand the actual cause while conducting educational campaigns and data-driven interventions tailored to address specific challenges.

This study also exhibited drinking water from tanks and other sources to have a higher likelihood of under-five mortality than piped water, suggesting that the source of drinking water may significantly impact child mortality rates, similar to studies exhibited in Kenya and Ethiopia [51, 52]. Piped water often undergoes regulated treatment from the dam, ensuring higher quality and reduced contamination, thus explaining the lower mortality rates associated with this source. Tank water, on the other hand, even if treated, is often stagnant, which can often favor microorganism growth, and its contamination may impact the entire community drinking water from that source instead of a household. The marginal significance of mortality when drinking water from rivers, lakes, and ponds could imply a potential risk, albeit not as pronounced as tank or non-piped sources, likely due to varying degrees of contamination and treatment levels. Investing in enhanced water infrastructure and extending access to treated, piped water could substantially decrease under-five mortality [55] linked to potentially contaminated sources like tanks while enforcing stringent water treatment for non-piped sources like rivers, lakes, and ponds could mitigate associated risks.

## Conclusion and recommendations

The analysis of determinants of under-five child mortality in Rwanda reveals significant associations with various maternal socio-demographic, child, and environmental factors. Maternal age, educational attainment, occupational status, wealth index, breastfeeding status, number of births, child’s sex, birth type, region, and drinking water source emerged as crucial determinants impacting under-five mortality. Addressing these factors through targeted interventions could significantly improve child survival rates in Rwanda. While some factors like maternal age, maternal education, and breastfeeding show protective effects, others, such as occupation, birth numbers, and environmental factors, pose increased risks, highlighting the multifaceted nature of under-five mortality.

Strategically tailored health initiatives targeting specific maternal education levels, occupational challenges, and regions with higher under-five mortality rates are crucial. Strengthening maternal and child health services to support breastfeeding practices, providing healthcare services tailored to different maternal age groups, and empowering mothers in high-risk groups for better caregiving practices are essential. Investing in water infrastructure to extend access to treated, piped water, reducing reliance on potentially contaminated sources, and enforcing stringent water treatment for non-piped sources would mitigate associated risks. Conducting region-specific interventions addressing disparities in healthcare access, infrastructure, and education, along with gender-sensitive programs addressing vulnerabilities in male children, is vital. Additionally, providing specialized care and attention for mothers carrying multiple pregnancies would effectively reduce under-five mortality and enhance child well-being in Rwanda.

### Strengths of the study

This study included a comprehensive approach, leveraging a substantial dataset of over 10,000 children under five years, enabling a multifaceted analysis of determinants influencing under-five mortality. This study used the birth recode data, which involved all birth histories of all interviewed mothers included in the study, and involved statistical rigor of multiple filters followed by association analysis and utilization of both univariate and multivariable analyses to enhance the reliability of the findings. Moreover, the study’s relevance to policy and practice stands out, as its insights into maternal age, education, occupation, wealth index, and environmental factors directly inform potential interventions and policy development in maternal and child health in Rwanda. It also contributes to existing knowledge by affirming established associations and uncovering potential new determinants of under-five mortality.

### Limitations of the study

Despite its strengths, this research faces limitations that are typical of similar studies. While it establishes associations between various factors and under-five mortality, it cannot definitively prove causation due to the nature of its design. Data collection might be affected by biases, including recall bias in maternal interviews, potentially impacting the accuracy of reported information. Additionally, the study might lack certain crucial variables that could significantly impact under-five mortality, such as specific health conditions, detailed access to healthcare facilities, or information regarding the quality of healthcare services. Addressing these limitations through more comprehensive data collection methods, including a broader array of variables, and a more nuanced study design would strengthen future research and deepen the understanding of under-five mortality in Rwanda and potentially in other similar contexts.

## References

[1] Mehmet T., Under-Five Mortality Causes and Prevention, in Mortality Rates in Middle and Low-Income Countries, Umar B, Editor. 2021, IntechOpen: Rijeka. p. Ch. 3.

[2] Black R.E., Levin C., Walker N., Chou D., Liu L., and Temmerman M., Reproductive, maternal, newborn, and child health: key messages from Disease Control Priorities 3rd Edition. Lancet, 2016. 388(10061): p. 2811–2824. doi: 10.1016/S0140-6736(16)00738-8 27072119

[3] Organization W.H. Child mortality and causes of death. 2023 [cited 2023 14 November]; Available from: https://www.who.int/data/gho/data/themes/topics/topic-details/GHO/child-mortality-and-causes-of-death.

[4] Sharrow D., Hug L., You D., Alkema L., Black R., Cousens S., et al., Global, regional, and national trends in under-5 mortality between 1990 and 2019 with scenario-based projections until 2030: a systematic analysis by the UN Inter-agency Group for Child Mortality Estimation. The Lancet Global Health, 2022. 10(2): p. e195–e206. doi: 10.1016/S2214-109X(21)00515-5 35063111 PMC8789561

[5] Estimation U.N.I.a.G.f.C.M-, Levels & trends in child mortality: report 2022. 2023, United Nations Children’s Fund (UNICEF) New York: New York.

[6] Sankar M.J., Natarajan C.K., Das R.R., Agarwal R., Chandrasekaran A., and Paul V.K., When do newborns die? A systematic review of timing of overall and cause-specific neonatal deaths in developing countries. Journal of perinatology: official journal of the California Perinatal Association, 2016. 36 **Suppl** 1: p. S1–S11. 10.1038/jp.2016.27 PMC4848744 27109087

[7] Tibebu N.S., Emiru T.D., Tiruneh C.M., Nigat A.B., Abate M.W., Getu B.D., et al., Potential determinant factors of under-five mortality in the Amhara region of Ethiopia. BMC Pediatr, 2022. 22(1): p. 205. doi: 10.1186/s12887-022-03253-x 35418057 PMC9008908

[8] Van Malderen C., Amouzou A., Barros A.J.D., Masquelier B., Van Oyen H., and Speybroeck N., Socioeconomic factors contributing to under-five mortality in sub-Saharan Africa: a decomposition analysis. BMC Public Health, 2019. 19(1): p. 760. doi: 10.1186/s12889-019-7111-8 31200681 PMC6570834

[9] CME Info—Child Mortality Estimates, in childmortality.org. 2023.

[10] UNICEF. Levels & trends in child mortality:report 2012. 2012 [cited 2023 27 November 2023]; Available from: https://data.unicef.org/resources/levels-trends-child-mortality-report-2012/.

[11] Farmer P.E., Nutt C.T., Wagner C.M., Sekabaraga C., Nuthulaganti T., Weigel J.L., et al., Reduced premature mortality in Rwanda: lessons from success. Bmj, 2013. 346: p. f65. doi: 10.1136/bmj.f65 23335479 PMC3548616

[12] Logie D.E., Rowson M., and Ndagije F., Innovations in Rwanda’s health system: looking to the future. Lancet, 2008. 372(9634): p. 256–61. doi: 10.1016/S0140-6736(08)60962-9 18619670

[13] Lu C., Chin B., Lewandowski J.L., Basinga P., Hirschhorn L.R., Hill K., et al., Towards universal health coverage: an evaluation of Rwanda Mutuelles in its first eight years. PLoS One, 2012. 7(6): p. e39282. doi: 10.1371/journal.pone.0039282 22723985 PMC3377670

[14] Rwanda U., Maternal Mortality Reduction Programme in Rwanda. 2011: p. 8.

[15] Gupta N., Hirschhorn L.R., Rwabukwisi F.C., Drobac P., Sayinzoga F., Mugeni C., et al., Causes of death and predictors of childhood mortality in Rwanda: a matched case-control study using verbal social autopsy. BMC Public Health, 2018. 18(1): p. 1378. doi: 10.1186/s12889-018-6282-z 30558586 PMC6296058

[16] Benimana C., Small M., and Rulisa S., Preventability of maternal near miss and mortality in Rwanda: A case series from the University Teaching Hospital of Kigali (CHUK). PLoS One, 2018. 13(6): p. e0195711. doi: 10.1371/journal.pone.0195711 29944664 PMC6019403

[17] Amoroso C.L., Nisingizwe M.P., Rouleau D., Thomson D.R., Kagabo D.M., Bucyana T., et al., Next wave of interventions to reduce under-five mortality in Rwanda: a cross-sectional analysis of demographic and health survey data. BMC Pediatr, 2018. 18(1): p. 27. doi: 10.1186/s12887-018-0997-y 29402245 PMC5799916

[18] Acheampong M., Ejiofor C., Salinas-Miranda A., Wall B., and Yu Q., Priority setting towards achieving under-five mortality target in Africa in context of sustainable development goals: an ordinary least squares (OLS) analysis. Glob Health Res Policy, 2019. 4: p. 3. doi: 10.1186/s41256-019-0108-0 31304284 PMC6599522

[19] National Institute of Statistics of Rwanda, Demographic and Health Survey 2019/2020—Final report. July 20, 2020.

[20] Sserwanja Q., Gatasi G., and Musaba M.W., Evaluating continuum of maternal and newborn healthcare in Rwanda: evidence from the 2019–2020 Rwanda demographic health survey. BMC Pregnancy and Childbirth, 2022. 22(1): p. 781. doi: 10.1186/s12884-022-05109-9 36261801 PMC9583497

[21] Sayinzoga F., Lundeen T., Gakwerere M., Manzi E., Nsaba Y.D.U., Umuziga M.P., et al., Use of a facilitated group process to design and implement a group antenatal and postnatal care program in Rwanda. Journal of Midwifery & Women’s Health, 2018. 63(5): p. 593–601. doi: 10.1111/jmwh.12871 30251304 PMC6220997

[22] Yaya S., Bishwajit G., Okonofua F., and Uthman O.A., Under five mortality patterns and associated maternal risk factors in sub-Saharan Africa: A multi-country analysis. PLoS One, 2018. 13(10): p. e0205977. doi: 10.1371/journal.pone.0205977 30359408 PMC6201907

[23] Fikru C., Getnet M., and Shaweno T., Proximate Determinants of Under-Five Mortality in Ethiopia: Using 2016 Nationwide Survey Data. Pediatric Health Med Ther, 2019. 10: p. 169–176. doi: 10.2147/PHMT.S231608 31908566 PMC6925557

[24] Liwin L.K. and Houle B.J.D.R., The effects of household and community context on mortality among children under five in Sierra Leone. 2019. 40: p. 279–306.

[25] Morakinyo O.M. and Fagbamigbe A.F., Neonatal, infant and under-five mortalities in Nigeria: An examination of trends and drivers (2003–2013). PLoS One, 2017. 12(8): p. e0182990. doi: 10.1371/journal.pone.0182990 28793340 PMC5549979

[26] Koroma M.M., Kabba J.A., Wanda J., Yu J., Zhou F., Liang Z., et al., Under-five mortality in Sierra Leone and possible associated factors: evidence from the 2019 Demographic and Health Survey. Health Policy Plan, 2022. 37(10): p. 1210–1220. doi: 10.1093/heapol/czac070 36052949

[27] Mosley W.H., Chen L.C.J.P., and d. review, An analytical framework for the study of child survival in developing countries. 1984. 10: p. 25–45. PMC2572391 12756980

[28] Kanmiki E.W., Bawah A.A., Agorinya I., Achana F.S., Awoonor-Williams J.K., Oduro A.R., et al., Socio-economic and demographic determinants of under-five mortality in rural northern Ghana. 2014. 14: p. 1–10. 10.1186/1472-698X-14-24 PMC4144693 25145383

[29] Ettarh R.R. and Kimani J., Determinants of under-five mortality in rural and urban Kenya. Rural Remote Health, 2012. 12: p. 1812. 22417123

[30] Ahinkorah B.O., Under-5 mortality in sub-Saharan Africa: is maternal age at first childbirth below 20 years a risk factor? BMJ Open, 2021. 11(9): p. e049337. doi: 10.1136/bmjopen-2021-049337 34593494 PMC8487196

[31] Ribeiro F.D., Ferrari R.A., Sant’Anna F.L., Dalmas J.C., and Girotto E., [Extremes of maternal age and child mortality: analysis between 2000 and 2009]. Rev Paul Pediatr, 2014. 32(4): p. 381–8. 25511003 10.1016/j.rpped.2014.05.002 PMC4311793

[32] Akinyemi J.O., Solanke B.L., and Odimegwu C.O., Maternal Employment and Child Survival During the Era of Sustainable Development Goals: Insights from Proportional Hazards Modelling of Nigeria Birth History Data. Ann Glob Health, 2018. 84(1): p. 15–30. doi: 10.29024/aogh.11 30873781 PMC6748258

[33] Antai D., Inequitable childhood immunization uptake in Nigeria: a multilevel analysis of individual and contextual determinants. BMC Infect Dis, 2009. 9: p. 181. doi: 10.1186/1471-2334-9-181 19930573 PMC2787508

[34] Aheto J.M.K., Predictive model and determinants of under-five child mortality: evidence from the 2014 Ghana demographic and health survey. BMC Public Health, 2019. 19(1): p. 64. doi: 10.1186/s12889-019-6390-4 30642313 PMC6332681

[35] Tagoe E.T., Agbadi P., Nakua E.K., Duodu P.A., Nutor J.J., and Aheto J.M.K., A predictive model and socioeconomic and demographic determinants of under-five mortality in Sierra Leone. Heliyon, 2020. 6(3): p. e03508–e03508. doi: 10.1016/j.heliyon.2020.e03508 32181389 PMC7063153

[36] Khan J.R. and Awan N., A comprehensive analysis on child mortality and its determinants in Bangladesh using frailty models. Archives of Public Health, 2017. 75(1): p. 58. doi: 10.1186/s13690-017-0224-6 28912949 PMC5588744

[37] Nattey C., Masanja H., and Klipstein-Grobusch K., Relationship between household socio-economic status and under-five mortality in Rufiji DSS, Tanzania. Glob Health Action, 2013. 6: p. 19278. doi: 10.3402/gha.v6i0.19278 23364083 PMC3556682

[38] Akinyemi J.O., Odimegwu C.O., Banjo O.O., and Gbadebo B.M., Clustering of infant deaths among Nigerian women: investigation of temporal patterns using dynamic random effects model. Genus, 2019. 75(1): p. 1–18.

[39] Azuike E., Onyemachi P., Amah C., Okafor K., and Anene J., Determinants of under-five mortality in South-Eastern Nigeria. J Community Med Public Health Care, 2019. 6: p. 049.

[40] Jacdonmi I., Suhainizam M., and Jacdonmi G., Breastfeeding, a child survival strategy against infant mortality in Nigeria. Current Science, 2016: p. 1282–1287.

[41] Meek J.Y. and Noble L., Policy Statement: Breastfeeding and the Use of Human Milk. Pediatrics, 2022. 150(1). doi: 10.1542/peds.2022-057988 35921640

[42] Bowatte G., Tham R., Allen K., Tan D., Lau M., Dai X., et al., Breastfeeding and childhood acute otitis media: a systematic review and meta‐analysis. Acta Paediatrica, 2015. 104: p. 85–95. doi: 10.1111/apa.13151 26265016

[43] Sankar M.J., Sinha B., Chowdhury R., Bhandari N., Taneja S., Martines J., et al., Optimal breastfeeding practices and infant and child mortality: a systematic review and meta‐analysis. Acta paediatrica, 2015. 104: p. 3–13. doi: 10.1111/apa.13147 26249674

[44] Rachmawati P.D., Kurnia I.D., Asih M.N., Kurniawati T.W., Krisnana I., Arief Y.S., et al., Determinants of under-five mortality in Indonesia: A nationwide study. Journal of Pediatric Nursing, 2022. 65: p. e43–e48. doi: 10.1016/j.pedn.2022.02.005 35216837

[45] Yemane G.D., The factors associated with under-five mortality in Ethiopia. Ann Med Surg (Lond), 2022. 79: p. 104063. doi: 10.1016/j.amsu.2022.104063 35860052 PMC9289410

[46] Bedada D., Determinant of under-five child mortality in Ethiopia. American Journal of Theoretical and Applied Statistics, 2017. 6(4): p. 198–204.

[47] Waldron I., Sex differences in infant and early childhood mortality: major causes of death and possible biological causes Too young to die genes or gender. 1998 New York United Nations, Department of Economic and Social Affairs. Population Division: p. 64–83.

[48] Waldron I., What do we know about causes of sex differences in mortality? A review of the literature. Popul Bull UN, 1985(18): p. 59–76. 12314310

[49] Zewudie A.T., Gelagay A.A., and Enyew E.F., Determinants of Under-Five Child Mortality in Ethiopia: Analysis Using Ethiopian Demographic Health Survey, 2016. Int J Pediatr, 2020. 2020: p. 7471545. doi: 10.1155/2020/7471545 33029153 PMC7527934

[50] Mekonnen D., Infant and child mortality in Ethiopia. The role of socioeconomic, demographic and biological factors in the previous, 2011. 5.

[51] Yemane G.D., Determinant factors of under-five mortality in rural Ethiopia. Ann Med Surg (Lond), 2022. 81: p. 104371. doi: 10.1016/j.amsu.2022.104371 36147140 PMC9486556

[52] Mutunga C.J., Environmental determinants of child mortality in Kenya, in Health inequality and development. 2011, Springer. p. 89–110.

[53] Gurmu E. and Mturi A.J., Trends and determinants of under-five mortality in Ethiopia: could the MDG four be met? Southern African Journal of Demography, 2014. 15(1): p. 49–80.

[54] Reilly M., Health Disparities and Access to Healthcare in Rural vs. Urban Areas. Theory in Action, 2021. 14(2).

[55] Wang L. and Jacoby H., Environmental determinants of child mortality in rural China: a competing risks approach. Vol. 3241. 2003: World Bank Publications.

